# Molecular Dynamics Simulation of Laser Induced Heating of Silicon Dioxide Thin Films

**DOI:** 10.3390/nano11112986

**Published:** 2021-11-06

**Authors:** Fedor Vasilievich Grigoriev, Vladimir Borisovich Sulimov, Alexander Vladimirovich Tikhonravov

**Affiliations:** 1 Research Computing Center, M.V. Lomonosov Moscow State University, Leninskie Gory, 119234 Moscow, Russia; vs@dimonta.com (V.B.S.); tikh@srcc.msu.ru (A.V.T.); 2 Moscow Center for Fundamental and Applied Mathematics, M.V. Lomonosov Moscow State University, Leninskie Gory, 119234 Moscow, Russia

**Keywords:** thin films, molecular dynamics, silicon dioxide films, laser induced damage

## Abstract

The full-atomistic classical molecular dynamics simulation of the laser heating of silicon dioxide thin films is performed. Both dense isotropic films and porous anisotropic films are investigated. It is assumed that heating occurs due to nodal structural defects, which are currently considered one of the possible causes of laser induced damage. It is revealed that heating to a temperature of 1000 K insignificantly affects the structure of the films and the concentration of point defects responsible for the radiation absorption. An increase in the heating temperature to 2000 K leads to the growth of the concentration of these defects. For “as deposited” films, this growth is greater in the case of a porous film deposited at a high deposition angle. Annealing of film reduces the difference in the concentration of laser induced defects in dense and porous films. The possible influence of optical active defects arising due to heating on the laser induced damage threshold is discussed.

## 1. Introduction

One of the key problems limiting the development of high energy laser systems is laser induced damage (LID) in thin transparent films of optical coatings [1,2,3]. LID is due to the loss of films transparency with an increase in laser impulse power, which leads to an explosive increase in the coating temperature and the subsequent destruction of its structure. To date, theoretical LID models can be divided into two classes: intrinsic and extrinsic [1,4]. Intrinsic models are applied to femtosecond laser pulses with extremely high power. These models take into account the properties of the film materials, such as band-gap values, refractive indices, and so on. The extrinsic models consider defects of structure as the cause of a sharp increase in laser radiation absorption with increasing laser power. These models describe LID from nanoseconds and longer pulses. The difference in the models is based on the experimental observed peculiarities of LID from short and long pulses [5,6].

Initially, the models describing LID were based on continual approaches including heat equations, kinetic equation, Boltzman equation, and so on [1,7,8,9]. In recent decades, due to significant progress in high-performance computing, it has become possible to apply the methods of atomistic simulation for studying the laser radiation interaction with condensed matter [10,11,12]. In the case of femtosecond laser pulses, for this simulation, the quantum methods are required to take into account the probabilities of many-photon absorption, electron tunneling in a strong electric field, and other quantum processes affecting LID [4]. The classic methods of atomistic simulation, such as molecular dynamics (MD), can be applied to study the effect of long pulses on the film structure. In this case, the interaction of laser radiation with electronic subsystem is taken into account through the simulation parameters.

In this work, the MD simulation of laser heating of silicon dioxide thin films is performed. It is assumed that heating occurs due to the amplification of laser radiation by the nodular defects appearing during the deposition process [13,14,15]. These structural defects are currently considered one of the possible causes of laser induced damage [16,17,18]. In [16], the effect of various types of nodules in the Ta_2_O_5_/SiO_2_ coatings on the local absorption of the laser radiation was studied. It was found that this absorption significantly depends on the boundaries of the nodules. In [17], the amplification of the electric field near nodular defects for multilayer mirrors was studied theoretically. This amplification can lead to an increase in the absorption of laser radiation, which affects the value of the LID threshold. In a review paper [18], nodular defects are considered to be the cause of laser induced damage to various types of coatings.

In this paper, we show that MD simulation can offer a description of laser-induced effects near nodular defects at the microscopic level. Both dense films and anisotropic films with high porosity are studied. The temperature and density distributions in the heated area are obtained for different pulse durations. The dependence of the concentration of point defects acting on the radiation absorption on the temperature of the heated area and the pulse duration is investigated. The differences in the effect of laser heating on the structure of as-deposited and annealed films are studied. The results obtained are discussed in terms of the possible causes of extrinsic LID.

## 2. Simulation Method

**Figure 1:**
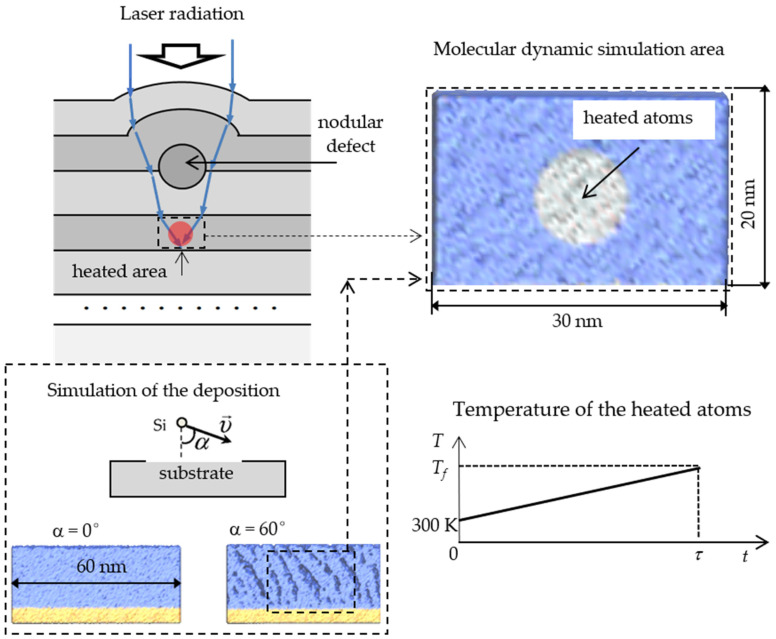
Scheme for modeling laser induced thin film heating.

The nodular defects in transparent media can focus laser radiation [19], which leads to heating of some portion of the film (see Figure 1). We assume that the temperature of the heated area increases during the entire duration of the pulse. Indeed, the characteristic time τ_h_ of thermal energy transfer from the heated area to the substrate can be estimated from the heat equation as τ_h_ ~ *c*ρ*L*^2^/χ, where the values of the specific heat capacity, mass density, and heat conductivity of silicon dioxide are equal to *c* = 10^3^ J/(kg·K), *ρ* = 2.2 × 10^3^ kg/m^3^, and *χ* = 2.0 W/(m·K) [20]. Since the characteristic value of film thickness *L* is about 10^−6^ m, we obtain *τ_h_* ~ 10^−6^ s. This value is much longer than the laser pulse duration; therefore, the stationary regime is not achieved during heating.

Heating of the film is modeled as atomistic clusters having dimensions of 30 × 20 × 20 nm with a total number of atoms of about 6 × 10^5^. The heated area inside the cluster is represented by a sphere with a radius of 5 nm centered in the middle of the cluster. The temperature of atoms inside the sphere is increased linearly from the room temperature 300 K to the final temperature *T_f_* during the pulse duration τ (Figure 1). The initial temperature of atoms outside the sphere is also 300 K, but after the start of heating is not controlled by the thermostat and depends on the heat flux from the heated area. The energy of interatomic interactions is calculated in the frame of the DESIL force field [21]:

*U* = *q_i_q_j_/r_ij_* + *A_ij_/r_ij_*^12^ − *B_ij_/r_ij_*^6^ (1)

where *q_i_*_(*j*)_ is the charge of the *i*(*j*)-th atom, *q*_O_ = −0.5*q*_Si_ = −0.65e, *A_ij_* and *B_ij_* are parameters of the Lennard-Jones potential for the van der Waals interaction, *r_ij_* is the interatomic distance, *A*_SiO_ = 4.6·× 10^−8^ kJ·(nm)^12^/mol, *A*_SiSi_ = *A*_OO_ = 1.5·× 10^−6^ kJ·(nm)^12^/mol, *B*_SiO_ = 4.2·× 10^−3^ kJ·(nm)^6^/mol, and *B*_SiSi_ = *B*_OO_ = 5·× 10^−5^ kJ·(nm)^6^/mol. The step of the molecular dynamic simulation is taken equal to 0.5 fs. Periodic boundary conditions are applied in all directions. To ensure the relaxation of the system volume under the heating conditions, the *NPT* (constant number of particles, pressure, and temperature) ensemble with Berendsen barostat [22] is used. The value of pressure applied to the boundary of the simulation box is 1 atm.

The atomistic clusters for simulating heating are prepared from the clusters of the silicon dioxide films obtained in our previous work using step-by-step MD procedure [23,24]. In [25], these clusters were used to study thermal stresses arising in thin films due to their heating as a whole. In this work, we study the effects associated with heating in nanoscale regions of atomistic clusters caused by nanoscale nodular defects focusing laser radiation. The simulation of heating is performed with clusters obtained at an energy of incoming Si atoms of 10 eV, which corresponds to the high-energy deposition. Two clusters, deposited at angles α = 0 and 60° are chosen. This is due to the fact that the film structure essentially depends on the deposition angle. Normal deposition at α = 0 produces dense and homogenous structures, while deposition at α = 60° leads to the formation of anisotropic and highly porous films [26].

All simulations are carried out using the GROMACS program [27], installed on the supercomputer “Lomonosov-2” of the HPC computing resources at Lomonosov Moscow State University [28].

## 3. Results and Discussion

**Figure 2:**
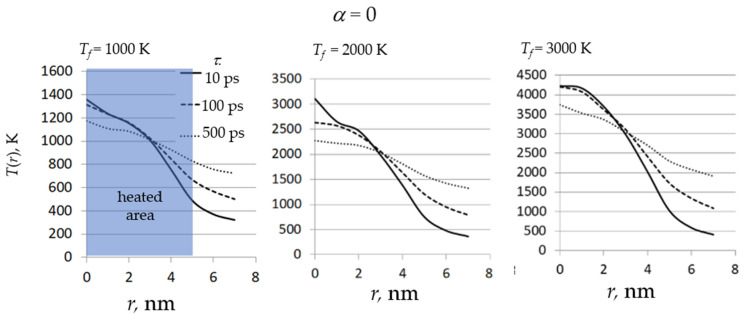
Dependence of the temperature *T* on the distance from the center of the heated area *r*, where *τ* is the heating time, *T_f_* is the final temperature, and α is the deposition angle.

**Figure 3:**
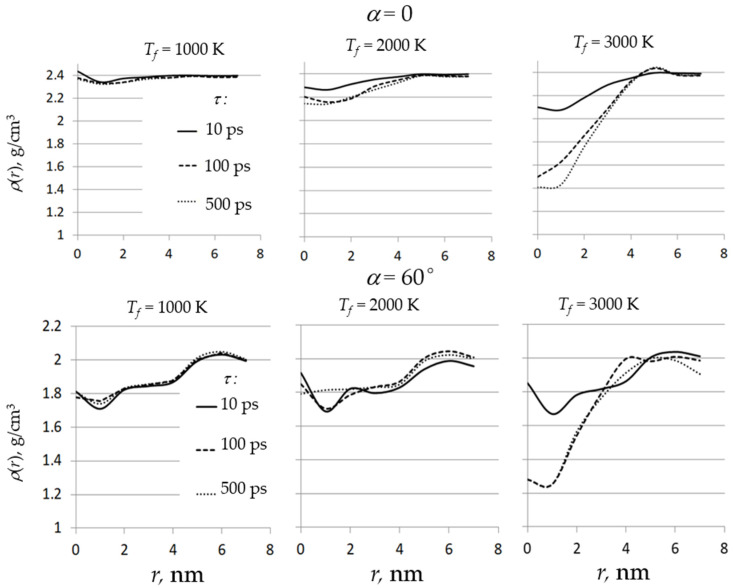
Dependence of the density ρ on the distance from the center of the heated area *r*, where *τ* is the heating time, *T_f_* is the final temperature, and α is the deposition angle.

**Figure 4:**
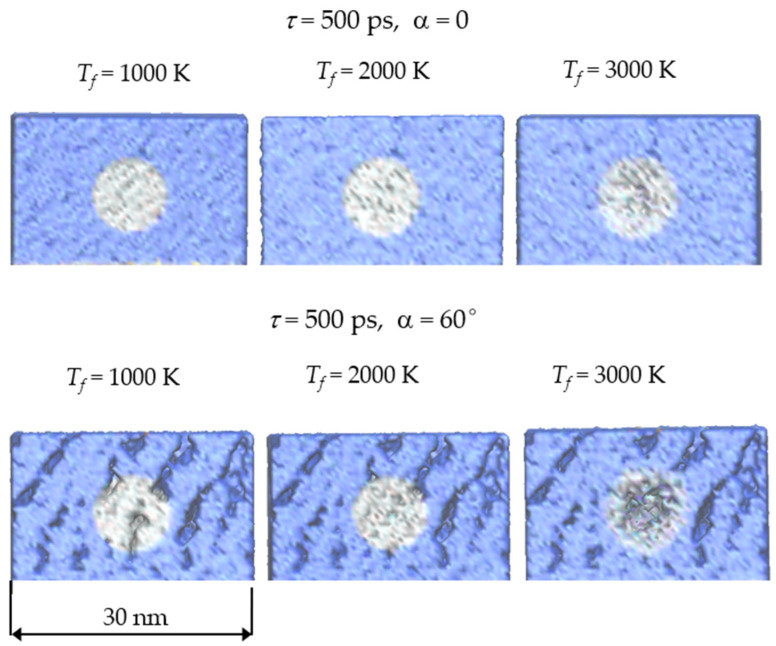
The final structures of the heated clusters for different values of the temperature *T_f_* and deposition angle α, where *τ* is the heating time.

The simulation results are shown in Figure 2, Figure 3 and Figure 4 and Table 1. In this work, the temperature of the heated area at the end of heating *T_f_* is taken equal to 1000 K, 2000 K, and 3000 K. The minimum value of *T_f_* is close to the temperatures that are used in the thin film annealing after deposition [29], and *T_f_* = 2000 K and 3000 K are about the melting and boiling points of quartz, respectively [30].

The heating time τ is taken to be 10 ps, 100 ps, and 500 ps. A 10 ps value corresponds to short laser pulses with durations from the hundreds of femtoseconds to several picoseconds. It should be taken into account that the laser irradiation interacts with the electron sub-system of matter. The characteristic time of transfer of absorbed energy from the electron sub-system to the nuclei is about several picoseconds [4]. Thus, even for femtosecond laser pulses, the heating time is several picoseconds. In the cases of *τ* = 100 ps and 500 ps, the heating time can be regarded as the laser pulse duration. Laser pulses with this duration are considered to be long, since their times are much longer than the time of energy transfer from the electron sub-system to the nuclei [4].

The temperature distributions over clusters are shown in Figure 2. To calculate the *T*(*r*) values, the average kinetic energy of all atoms between the spheres with radii *r* and *r* + ∆*r*, where ∆*r* = 1 nm is calculated and inverted into temperature.

As can be seen from the plots in Figure 2, in all cases the temperature decreases monotonically with increasing distance to the center of the heated area. The difference in the temperature of atoms inside and outside of the heated area decreases with an increase in the laser pulse duration. Even at *τ* = 500 ps, the temperature distributions are far from uniform. In the case of the *T_f_* = 3000 K, the temperature at *r* < 3 nm exceeds the quartz boiling point, which leads to partial evaporation of the film material, which is confirmed by visualization of the structures (see Figure 4). Since there are no noticeable differences in the temperature distributions in the case of α = 60° compared to α = 0°, the data are presented only for the normally deposited film.

The density distributions ρ (*r*) are shown in Figure 3. The ρ (*r*) is equal to the density inside the area between spheres with radii *r* and *r* + ∆*r*, where ∆*r* = 1 nm. The density profiles at heating temperature *T_f_* = 1000 K change insignificantly with increasing heating time τ and correspond to the profiles of the unheated films. The difference in the density of films deposited at α = 0 and α = 60° (see the upper and lower plots in the left column in Figure 3) is caused by the difference in the deposition angles and is not related to heating.

An increase in the heating temperature to *T_f_* = 2000 K leads to changes in the density profiles of the most heated region near *r* = 0. In a dense film (α = 0), the density decreases essentially with increasing heating time from *τ* = 10 ps to *τ* = 100 ps. A further increase of τ to 500 ps has no significant effect on the density profile. In the case of a porous SiO_2_ film (α = 60°), heating to *T_f_* = 2000 K for 500 ps leads to smoothing of the density profiles at *r* < 2 nm. Visual analysis reveals that this heating leads to the disappearance of the large pore in the center of the heated area (see the bottom row in Figure 4).

An increase in the heating temperature to *T_f_* = 3000 K leads to the sharp decrease in the density near the center of the heated area for both films. This is due to the start of the boiling process, since the temperature near the center of the heated area exceeds the quartz boiling point.

Heating of the films can lead to an increase in the concentration of point defects, such as oxygen-deficient silicon Si_3_ and nonbridging oxygen center O_1_. The experimentally observed absorption and luminescence at photon energies below the band-gap of silica is attributed to these defects [31,32]. Thus, an increase in the concentration of these defects leads to an increase in absorption, which increases the heating of the film. Ultimately, this can lead to a thermal explosion destroying the structure.

The values of the point defects concentration are presented in Table 1. The concentration of O_1_ and Si_3_ centers in the “as deposited” films at room temperature at α = 60° is almost twice as high as at α = 0. This difference is explained by the high porosity of the film deposited at α = 60°, which leads to an increase in the concentration of the defects. As can be seen from the data in Table 1, this noticeable difference persists under heating.

Heating to *T_f_* = 1000 K insignificantly affects the concentration of O_1_ and Si_3_ centers. An increase in the final value of the heating temperature to *T_f_* = 2000 K leads to a noticeable increase in the concentration of defects, primarily of Si_3_ centers. An increase in the heating time from τ = 100 ps to τ = 500 ps at *T_f_* = 2000 K also leads to an increase in the defects concentration. For a normally deposited film, heating to *T_f_* = 3000 K at τ = 500 ps is accompanied by an increase in c(O_1_) and c(Si_3_) by approximately four and twenty times, respectively, compared with the values in the film at room temperature. In the case of α = 60°, the relative increase in the of c(O_1_) and c(Si_3_) values caused by heating at *T_f_* = 3000 K and *τ* = 500 ps is slightly higher than in the case of a normally deposited film. 

Annealing of the films significantly affects the c(O_1_) and c(Si_3_) values (see the lower part of Table 1). In this work, annealing is simulated as described in [33] at an annealing temperature of 1300 K during 1 ns. As expected, annealing reduces the c(O_1_) and c(Si_3_) values. This decrease is more noticeable for a porous film deposited at α = 60°. On the whole, the tendency towards an increase in the defects concentration with an increase in the heating temperature and heating time is retained for the annealed films. At the same time, preliminary annealing of the films reduces the differences in c(O_1_) and c(Si_3_) values for the films deposited at α = 60° and α = 0. For instance, c(O_1_) = 2.46 (α = 0°) and c(O_1_) = 2.55 (α = 60°) at *T_f_* = 2000 K and *τ* = 500 ps.

Let us discuss the obtained results from the point of view of the LID problem. Heating to an average temperature *T_f_* = 1000 K insignificantly affects the structure of the films, including the concentration of point defects. For this reason, this heating does not initiate LID. This is true both for a normally deposited dense film and for a highly porous film deposited at α = 60°.

Heating to *T_f_* = 2000 K is accompanied by a noticeable increase in the concentration of optical active defects. This growth is observed at all values of the laser pulse duration. This leads to an increase in the radiation absorption, which is accompanied by an increase in the film temperature, which ultimately can lead to a thermal explosion. Thus, this heating can initiate LID. In the case of “as deposited” films, the concentration of the laser induced defects in porous film deposited at α = 60° increases more than in a normally deposited film. Thus, for porous films, the threshold value of LID can be lower.

Annealing the films before heating leads to a decrease in the difference of the defects concentration in films deposited at α = 0 and α = 60°, so the difference in the LID threshold for these films can also decrease. In addition, the inhomogeneity of the structure of the porous film can partially prevent the focusing of laser beam by nodular defects. For this reason, the heating temperature of a porous film can be lower than that for a dense film at the same values of the radiation power. Thus, the porous film can be even more LID-resistant than the dense film. An increase in the laser induced damage threshold with an increase in the deposition angle and film porosity was observed experimentally for SiO_2_ films [34,35]. To summarize, an increase in the concentration of point defects due to heating near the nodular defects can be noted. This increase depends on film structure. It is shown that post deposition annealing reduces the concentration of point defects, and thus can reduce the negative effect of nodular defects on the LID threshold.

## 4. Conclusions

In this work, the atomistic simulation of the laser induced heating of the silicon dioxide thin films is performed. Heating is caused by focusing the laser beam by the nodular defects in the film structure.

It is found that heating to 1000 K for 500 ps insignificantly affects the structure of the films and concentration of the optical active point defects. An increase in the heating temperature to 2000 K leads to an increase in the concentration of point defects, especially oxygen-deficient silicon Si_3_. For “as deposited” films, this increase is greater in the case of a porous film deposited at a large deposition angle. Annealing the films reduces the difference in the concentration of laser induced defects for dense and porous films.

## Figures and Tables

**Table 1:** Concentration of defects c(O_1_) and c(Si_3_) (%, lower index is equal to the coordination number) in films at different deposition and heating conditions, where *T_f_* is the final heating temperature, τ is the heating time, α increase s the deposition angle, and *T*_0_ is the film temperature before heating.

**“As Deposited” Films**						
**α**	**τ, ps**	**Defect**	***T*_0_ = 300 K** **(before Heating)**	***T_f_*, K**		
				**1000**	**2000**	**3000**
0	10	c(O_1_)	1.13	1.17	1.43	2.26
		c(Si_3_)	0.35	0.32	0.87	2.50
	100	c(O_1_)		1.16	1.44	2.70
		c(Si_3_)		0.30	0.91	3.54
	500	c(O_1_)		1.12	1.61	4.69
		c(Si_3_)		0.36	1.62	7.52
60	10	c(O_1_)	2.17	2.20	3.25	6.35
		c(Si_3_)	0.75	0.86	3.03	8.20
	100	c(O_1_)		2.19	3.53	8.40
		c(Si_3_)		0.90	3.82	12.05
	500	c(O_1_)		2.12	3.32	11.08
		c(Si_3_)		0.92	4.35	18.11
**Annealed Films**						
α	τ, ps	Defect	*T*_0_ = 300 K (before heating)	*T_f_*, K		
				1000	2000	3000
0	10	c(O_1_)	1.04	1.03	1.88	4.90
		c(Si_3_)	0.47	0.50	2.07	7.17
	100	c(O_1_)		1.05	2.29	7.40
		c(Si_3_)		0.50	2.87	11.38
	500	c(O_1_)		1.04	2.46	10.48
		c(Si_3_)		0.51	3.44	17.50
60	10	c(O_1_)	1.42	1.45	2.39	5.24
		c(Si_3_)	0.67	0.92	2.83	7.57
	100	c(O_1_)		1.36	2.58	6.99
		c(Si_3_)		0.89	3.46	10.63
	500	c(O_1_)		1.36	2.55	9.95
		c(Si_3_)		0.89	3.72	16.76

## Data Availability

Not applicable.
